# Microanatomical Study of Embryonic Gonadal Development in Japanese Quail (*Coturnix japonica*)

**DOI:** 10.1155/2014/168614

**Published:** 2014-09-03

**Authors:** Sittipon Intarapat, Orawan Satayalai

**Affiliations:** ^1^Department of Anatomy, Faculty of Science, Mahidol University, Bangkok 10400, Thailand; ^2^Department of Biology, Faculty of Science, Chulalongkorn University, Bangkok 10330, Thailand

## Abstract

Gonadal development of quail embryos was examined histologically using histological and histochemical methods. In the present study, quail embryos were studied at various stages of incubation period based on phases of gonadogenesis. Germ cell migration was observed on day 3-4 but gonadal differentiation and gonadal function were observed on day 6–8 and day 11–14, respectively. During germ cell migration, quail primordial germ cells (qPGCs) were successfully detected in both left and right genital ridges as well as the dorsal mesentery by lectin histochemistry. Unexpectedly, qPGCs-like cells were found next to the neural tube by Mallory-AZAN stain. During gonadal differentiation, embryonic sex can be distinguished histologically since day 8 of incubation. Embryonic testis exhibited a thin cortex, whereas embryonic ovary exhibited a thick cortex. Testicular cord formation was found in the medulla of embryonic testes while the lacunae and fat-laden cells were found in the medulla of embryonic ovary during gonadal function. This is the first report on a comparison of phases of gonadogenesis and histochemical study of quail embryonic gonads in both sexes.

## 1. Introduction

Avian embryos have become a favorable model in developmental biology and stem cell biology [1, 2]. Their reproductive organs have unique characteristics. In female, the gonads and accessory embryonic oviducts develop asymmetrically, whereas symmetrical gonads develop in male [3–5]. Recent evidence shows that even male embryos have a greater number of germ cells and pluripotent stem cells in the left gonad [6]. Quail embryos have several advantages as an animal model: the embryos take about 16 days to develop inside their eggs until hatching [7, 8] while chicken embryos take about 22 days [9], the eggs are available all year round [10, 11], and the embryonic development is well studied [7, 8, 12]. Moreover, quail embryos and their reproductive organs are also recommended for studying reproductive and development toxicology [8, 13–16].

Gonadal development of quail embryos is similar to other avian species. The process in which the embryonic gonads are generated is called “gonadogenesis” [18, 19]. Phase of such process is divided into three major events such as genital ridge formation, gonadal differentiation, and gonadal function [19]. In nonmammalian species, two molecular mechanisms underlying gonadogenesis including genetic cascade and sex determination have been reported [4, 5, 18–20]. In mammalian counterparts, sex determining region Y, SRY gene located on the Y chromosome was reported to play a role in testicular development [21]. Generally, in vertebrates, gonadogenesis begins with primordial germ cells (PGCs) migration [22, 23]. PGCs originate from the extraembryonic region (on the first day of gestation) and then migrate towards the genital ridges to settle down on this region [23–26]. Mammalian PGCs are derived from the endodermal cells of the yolk sac of the hindgut [24, 25]. On the contrary, avian PGCs originate from the central zone of blastodisc [23, 26–29] and then migrate anteriorly to extraembryonic region called “germinal crescent” [23, 26]. From such region, PGCs subsequently enter into the blood vessels and extravasate from the endothelium of capillaries towards the genital ridges by amoeboid movement [23, 26, 30, 31], using either pseudopodia or filopodia-like processes [26, 32]. The settled PGCs with contributions from the coelomic epithelium and the mesonephroi give rise to various cell types in the developing gonads [33–35]. In the developing gonads, PGCs and surrounding somatic cells initiate to form the bipotential (indifferent or undifferentiated) gonads [34–36]. Next, undifferentiated gonads enter into the process of sex determination on day 4 of gestation [4, 5, 18]. In male (ZZ) chicken embryos,* DMRT1* was reported to be testis-determining gene [5, 37], whereas* FOXL2* and* RSPO1 *were proposed as ovary-determining genes in female (ZW) chicken embryos [38, 39]. Finally, sexually differentiated gonads appear to become the mature gonads being able to produce the functional gametes. This process takes place in gonadal function under the regulation of steroid hormones [3, 19]. The objective of this study aimed to describe gonadal development of quail embryos in three major phases including genital ridge formation, gonadal differentiation, and gonadal function using histological and histochemical methods. This information will be useful for studying reproductive and developmental biology in avian species.

## 2. Materials and Methods

### 2.1. Animals and Embryo Collection

Japanese quail (*Coturnix japonica*) eggs were obtained from the Department of Animal Science, Kasetsart University. Quail eggs were incubated at 37.5°C in a humid atmosphere and automatically turned by the incubator. The eggs were collected on days 3, 4, 6, 8, 11, and 14 of incubation and the embryos were staged according to Padgett and Ivey, 1960 (P&I) [17]. Different stages of the embryos and their gonads were dissected under the SZ-PT stereomicroscope (Olympus, Japan). The embryonic gonads were fixed in Bouin's and Rossman's fluids for histological and histochemical studies, respectively.

### 2.2. Conventional Histochemical Methods

The fixed gonads were dehydrated in graded series of ethanol concentrations, cleared in xylene, and embedded in Paraplast (Sherwood Medical Company, St. Louis, MO, USA.). The embedded gonads were cut at 6 *μ*m with a microtome (The Gemmary, Fallbrook, USA). The sections were stained with hematoxylin and eosin.

For histochemical study, the sections were processed to Mallory-AZAN stain [40] for studying connective tissues in the developing gonads. The fixed sections were deparaffinized and hydrated. The sections were immersed in aniline blue and acid alcohol for 45 and 2 min, respectively. Next, the sections were stained in azocarmine for 1 hr and then rinsed in distilled water. The sections were differentiated in aniline alcohol and treated with acid alcohol for 2 min. The treated sections were transferred to phosphotungstic acid for 2 hr and rinsed with distilled water. The rinsed sections were stained in aniline blue for 1 hr and rinsed with distilled water and then treated with phosphotungstic acid for 3 min. Afterwards, the treated sections were rinsed in acidulated water and 70% ethanol. Finally, the rinsed sections were dehydrated in graded series of ethanol and mounted with the cover slips.

### 2.3. Lectin Histochemistry

To localize PGCs in 3-day-old quail embryos at the early phase of gonadogenesis, the sections were subjected to lectin histochemistry. WFA lectin (*Wisteria floribunda*, Biotin conjugate, Sigma-Aldrich, MO, USA) binding with its carbohydrate specificity (*α*/*β*-GalNAc) on quail germ cells [41, 42] was used in this study. The biotin-streptavidin method was applied for visualization of lectin binding (Chemicon International, USA). Briefly, endogenous peroxidase was blocked with 0.3% H_2_O_2_/methanol for 30 min at room temperature. Then, samples were incubated for 1 hr in humidified chamber at room temperature with a solution of biotinylated lectin diluted in 0.05 M PBS, pH 7.4 in a final concentration of 50 *μ*g/mL. Sections were washed in 0.05 M PBS, pH 7.4, and a solution of the biotin-streptavidin peroxidase complex was added following the manufacturer's instructions. Lectin binding sites were revealed using diaminobenzidine-hydrogen peroxide medium (DAB-H_2_O_2_) for 10 min in humidified chamber at room temperature. Background staining and nonspecific binding were blocked by preincubating the sections in a solution of 0.1% normal goat serum in PBS for 30 min in humidified chamber at room temperature. Control sections were incubated with the incubation medium omitting the biotinylated lectin. The sections were studied and photographed by PM-10 M3 camera (Olympus, Japan).

### 2.4. Statistical Analysis

To quantify the number of quail-PGCs (WFA positive cells) in the genital ridges, WFA positive cells were counted starting from the first section containing left and right genital ridges to the tenth section. To avoid counting the same cells more than once, one in three sections was counted until the last section of the genital ridges was reached. Unpaired Student's *t*-test with two-tailed distribution and two-sample unequal variance was used to compare (pairwise) the number of WFA positive cells between left and right sides of genital ridges. The quantitative data was presented as mean ± SD, which was analysed using SPSS.

## 3. Results

From anatomical observations (data not shown), the presumptive gonads (the genital ridges) locate at the medioventral part of the mesonephroi. Embryonic sex can be first distinguished by gonadal morphology on day 7 of incubation. Male embryos exhibit bilateral gonads, whereas female embryos exhibit asymmetrical gonads in which only the left gonad develops into a functional ovary. Embryonic accessory ducts including Wolffian and Müllerian duct can also be observed in male and female embryos, respectively.

According to phases of gonadogenesis described by Clinton and Haines, 2001, in the present study, gonadogenesis of quail embryos was studied in three phases such as genital ridge formation, gonadal differentiation, and gonadal function. The 3- and 4-day-old embryos were subjected to phase of genital ridge formation. While the 6- and 8-day-old embryos were subjected to phase of gonadal differentiation, the 11- and 14-day-old embryos were subjected to phase of gonadal function. The details of histological observations of gonadogenesis in each phase are described as follows.

**Figure 1:**
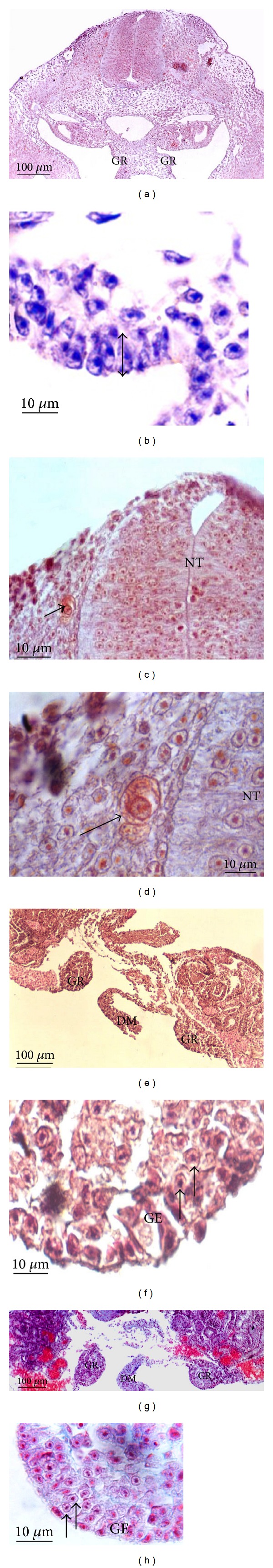
Photomicrographs of 3- and 4-day-old quail embryos sectioned during genital ridge formation. (a) The region of the genital ridges (GR) of 3-day-old embryos (AZAN ×40); (b) the genital ridge epithelium (double arrow) (H&E ×400); (c, d) qPGCs-like cells (arrow) located next to the neural tube (NT) (AZAN ×100, ×1000, resp.); (e) the region of the hindgut of 4-day-old embryos showing the genital ridges (GR) and the dorsal mesentery (DM) (H&E ×40); (f) qPGCs (arrows) settled in the genital ridge epithelium (GE) (H&E ×400); (g) the region of the hindgut of 4-day-old embryos showing the genital ridges (GR) and the dorsal mesentery (DM) (AZAN ×40); (h) qPGCs (arrows) settled in the genital ridge epithelium (GE) (AZAN ×400). Note: two layers of the genital ridge epithelium can be obviously seen by Mallory-AZAN stain.

*Phase of Genital Ridge Formation (Day 3-4 of Incubation).* On day 3, the genital (gonadal) ridges containing simple germinal epithelium were observed (Figures 1(a) and 1(b)). Unexpectedly, qPGCs-like cells were detected next to the neural tube by Mallory-AZAN (Figures 1(c) and 1(d)). On day 4, the proliferation of germinal epithelium was noticed by bulging out of the epithelium (Figures 1(e) and 1(g)). The settlement of germ cells in the germinal epithelium was found (Figures 1(f) and 1(h)). Germ cell can be distinguished from neighboring somatic cell by having larger cell with larger nucleus and clearer cytoplasm (Figure 1(h)). In addition, the thickness of germinal epithelium containing two layers of epithelia could be revealed by Mallory-AZAN due to the connective tissue underlying germinal epithelium which was stained blue (Figure 1(h)).

**Figure 2:**
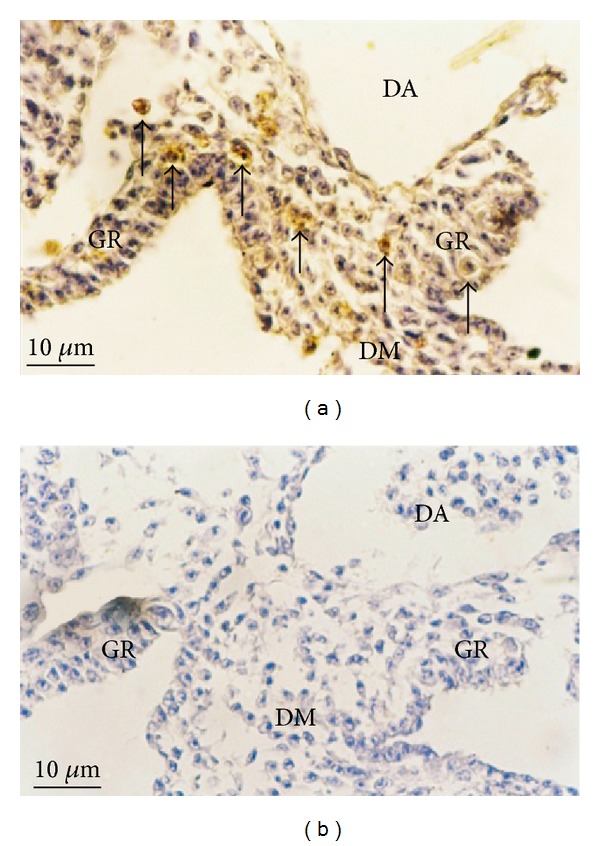
Photomicrographs of 3-day-old quail embryos sectioned during genital ridge formation. (a) qPGCs and WFA positive cells (arrows) were detected in both left and right genital ridges (GR) as well as the dorsal mesentery (DM), but not in the dorsal aorta (DA) (lectin HC ×400); (b) no WFA positive cells in the negative control (×400).

During genital ridge formation, quail primordial germ cells (qPGCs) can be seen in their migratory routes and the genital ridges. In this study, qPGCs were identified by lectin histochemistry in which WFA lectin from* Wisteria floribunda* was used as qPGCs marker. The result showed that WFA positive cells were detected in both left and right genital ridges as well as the dorsal mesentery (Figure 2(a)). No positive cells were detected in the negative control (Figure 2(b)). The number of qPGCs was counted as shown in Table 1. Total PGC numbers (WFA positive cells) in the left and right genital ridges were 1,496 (26 ± 7) and 1,382 (24 ± 9), (*p* = 0.1, *n* = 6) cells, respectively. There was no statistically significant difference in the average number of WFA positive cells between left and right genital ridges (Table 1).

**Figure 3:**
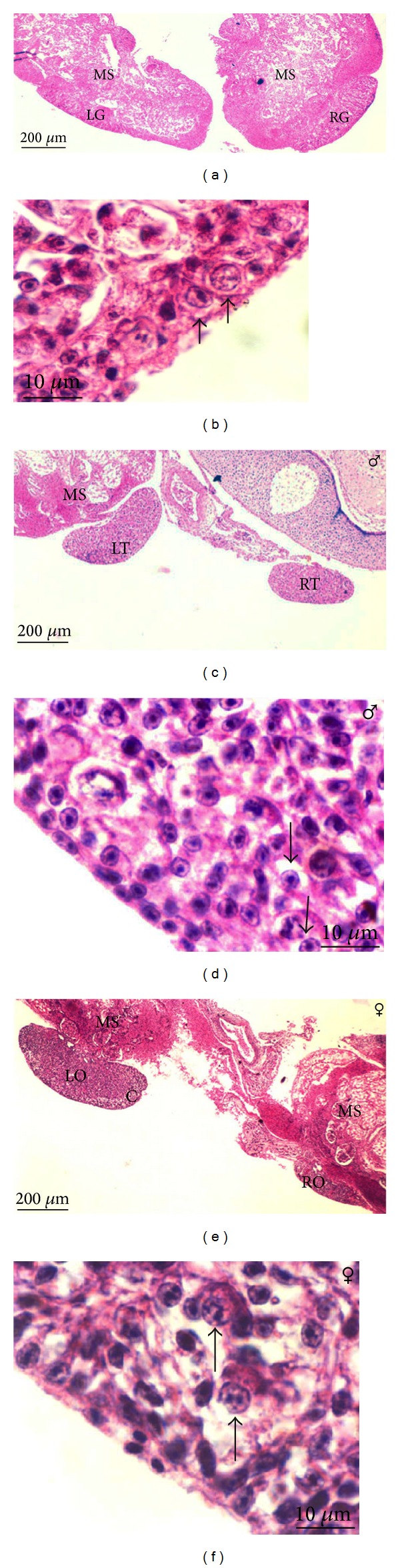
Photomicrographs of 6- and 8-day-old quail embryos sectioned during gonadal differentiation. (a) Six-day-old left and right indifferent gonads (LG, RG) are situated at the medioventral part of the mesonephroi (MS) (H&E ×40); (b) germ cells (arrows) were found in the germinal epithelium of indifferent gonads (H&E ×1000); (c) 8-day-old left and right embryonic testes (LT, RT) are situated at the medioventral part of the mesonephroi (MS) (H&E ×40); (d) male germ cells (arrows) were found in the medulla (H&E ×1000); (e) 8-day-old left and right embryonic ovaries (LO, RO) are situated at the medioventral part of the mesonephroi (MS) (H&E ×40); (f) female germ cells (arrows) were found in the cortex (H&E ×1000). Note: thick cortex (C) and asymmetrical gonads were observed in female embryos.

*Phase of Gonadal Differentiation (Day 6–8 of Incubation).* On day 6, embryonic sex cannot yet be distinguished by gonadal histology. Genital ridges are transformed into the rod-shaped structures called “indifferent gonads or undifferentiated gonads.” Both left and right indifferent gonads were situated at the medioventral part of the mesonephroi (Figure 3(a)). Germ cells were found in the germinal epithelium of indifferent gonads (Figure 3(b)). On day 8, gonadal differentiation can be observed and embryonic sex can be distinguished by gonadal histology. In male embryos, the indifferent gonads developed into bilateral testes and male germ cells were found in the embryonic testes (Figures 3(c) and 3(d)). In female embryos, gonadal asymmetry was observed since the left gonad only developed, whereas the right gonad regressed. The left embryonic ovary was much larger than the right one and female embryonic germ cells were detected in the developing ovaries (Figures 3(e) and 3(f)).

**Figure 4:**
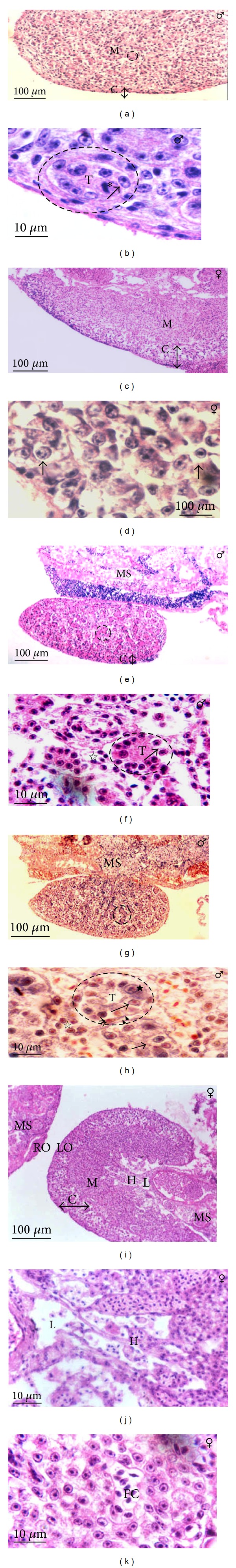
Photomicrographs of 11- and 14-day-old quail embryos sectioned during gonadal function. (a) 11-day-old embryonic testis exhibits thin cortex (c) (H&E ×200); (b) testicular cord (T, ellipse) containing spermatogonium (arrow) and Sertoli cell (asterisk) was found in the medulla (H&E ×400); (c) 11-day-old embryonic ovary exhibits thick cortex (C, double arrow) (H&E ×200); (d) oocytes-like germ cells (arrows) were found in the cortex (H&E ×400); (e) 14-day-old embryonic testis exhibits numerous testicular cords (circle) (H&E ×100); (f) testicular cords (T, circle) and interstitial cells (star) were observed in the medulla (H&E ×200); (g) 14-day-old embryonic testis exhibits numerous testicular cords (circle) (AZAN ×100); (h) spermatogonium (arrows) and Sertoli cell (asterisk) were found in testicular cord (T, circle), whereas peritubular myoid cell (double arrows) whose nucleus stained orange was found at the basement membrane (AZAN ×400). Note: the basement membrane of testicular cord (double arrowhead) stained by AZAN is more distinctive than that of testicular cord stained by H&E. (i) 14-day-old left and right embryonic ovaries (LO, RO) are situated at the medioventral part of the mesonephroi (MS) and thick cortex containing oocytes-like germ cells (C, double arrow) was noticed in left embryonic ovary (H&E ×100); (j) the lacunae (L) and hilum (H) were observed in the medulla of left embryonic ovary (H&E ×200); (k) the fat-laden cells (FC) were also observed in the medulla of left embryonic ovary (H&E ×400). Note: left embryonic ovary is much larger than right embryonic ovary. There is no difference between the cortex and medulla of right embryonic ovary.

*Phase of Gonadal Function (Day 11–14 of Incubation).* On day 11, gonadal function was first noticed by sex cord development. In male embryos, primary sex cord, the testicular cord was observed (Figures 4(a) and 4(b)). Furthermore, a thin cortex and spermatogonium were also observed in the developing testes (Figure 4(b)). In female embryos, the cortical cord containing oocytes-like germ cells was observed (Figures 4(c) and 4(d)). On day 14, the distinction of testicular cord in the developing testes can be indicated (Figures 4(e) and 4(g)). Obviously, the basement membrane of testicular cord containing male germ cells as well as other somatic cells (i.e., Sertoli cells and peritubular myoid cells) is well demarcated by Mallory-AZAN due to the underlying connective tissue which was stained deep blue (Figure 4(h)). Infiltration of the red blood cells, which was stained red as well as interstitial cells locating between the testicular cords, was also observed by Mallory-AZAN (Figure 4(h)). The difference between left and right embryonic ovaries can be manifested at this phase. The right ovary was observed as a vestigial structure, whereas a fan-shaped ovary was observed on the left side (Figure 4(i)). In addition, oocyte-like germ cells were found in the thick cortex (Figure 4(i)), but the lacunae (unfilled spaces) and the hilum (ovarian stalk connected with the mesonephroi) were found in the medulla (Figure 4(j)). Likewise, fat-laden cells were also found in the medulla (Figure 4(k)).

## 4. Discussion

This study revealed the details of major phases of gonadogenesis in quail embryos in both sexes based on histological and histochemical observations. Previous studies have merely described development of quail embryonic testis and ovary by H&E [43, 44]. Alternatively, lectin histochemistry and Mallory-AZAN stain were applied for identifying qPGCs as well as gonadal structures in our study. During genital ridge formation, putative qPGCs were found in both left and right genital ridges as well as the dorsal mesentery. The qPGCs in the genital ridges can be distinguished from neighboring somatic cells by having larger cells with larger nuclei and clearer cytoplasm by H&E. Similar studies reported that chicken PGCs can be easily distinguished by being larger cells containing a larger nucleus than surrounding somatic cells [45, 46], indicating common characteristics of avian PGCs. Identification of qPGCs was confirmed by WFA lectin histochemistry. Lectin from* Wisteria floribunda* (WFA) was reported to be a marker for qPGCs since it specifically reacted with its sugar-binding specificity,* α*/*β*-GalNAc on the surface of qPGCs [41, 42, 47]. The result showed that WFA positive cells were detected in the genital ridges as well as the dorsal mesentery, suggesting that qPGCs migrate through the dorsal mesentery on their migratory routes. This demonstrates that such lectin is specifically expressed on the surface of migrating-qPGCs during germ cell migration. Other studies reported that qPGCs aggregating on the blastodisc of young embryos migrated actively through the dorsal mesentery towards the genital ridges [27, 48]. Sugar-binding protein was reported to be expressed during avian germ cell migration [41, 42, 47, 49]. Additionally, quail PGCs were positive for WFA lectin, whereas chick PGCs were positive for* Griffonia simplicifolia* II (GS-II) lectin [42]. This suggests that there are differences in sugar-binding protein among avian species.

This study unexpectedly found that qPGCs-like cells were located adjacent to the neural tubes by Mallory-AZAN. Extragonadal distribution of avian germ cells was reported since 90% of the ectopic PGCs were found in the head, mainly in the mesenchyme surrounding the neural tube [50]. Our results suggested that those cells might be qPGCs based on the morphological criteria. However, identification of qPGCs at the extragonadal regions using definitive markers such as Vasa and QCR-1 requires further study.

In the phase of gonadal differentiation, embryonic sex can be first distinguished anatomically and histologically on days 7 and 8 of incubation, respectively. Histologically, the cortex of female developing gonads is much thicker than that of male developing gonads. We found that male developing gonad exhibited thin cortex, whereas female developing gonads exhibited thick cortex. In this regard, estrogen was reported to play a role in gonadal differentiation in avian embryos [3, 51, 52]. Several experiments demonstrated that* in ovo* injection of exogenous estrogens as well as phytoestrogens resulted in a thickening of the cortex in male quail gonads [10, 16, 53, 54], indicating that estrogen induces cell proliferation in the cortex of developing gonads. We also found that sexually differentiated gonads, on day 11 of gonadal differentiation, ultimately developed into the mature gonads on day 14 of gonadal function in both sexes. This event can be observed by the appearance of the primary sex cords that further develop into the secondary sex cords. In male (ZZ) chicken embryos, testicular cord development is regulated by testis-determining factor, DMRT1 produced by Z chromosome [4, 5, 20, 37]. Conversely, in female (ZW) chicken embryos, there was medullary cord degeneration in the medulla which resulted in secondary sex cord development giving rise to the thick cortex. Molecular studies proposed that FOXL2 and RSPO1 might be ovary-determining factors since their expressions were detected in the developing ovary [38, 39]. Our findings may support the theory of medullary versus cortical development in male and female embryonic gonads [55, 56]. Nevertheless, the molecular study of gene expression patterns of testis and ovary-determining genes in quail embryonic gonads is required.

During gonadal function, sexually differentiated gonads started to become the mature gonads. In male embryos, the structure of testicular cords of 14-day-old embryos was well delineated. The basement membrane of such cords was demarcated by Mallory-AZAN stain [40] and therefore this method is being used to study the formation of male cord as well as collagen fibers [57, 58]. The results showed that the connective tissue underlying testicular cord epithelium was stained deep blue, demonstrating extracellular materials of the cord. This suggests that such technique is advantageous for differentiating the boundary between testicular cords and extracellular components.

In female embryos, the left ovary showed gonadal growth by forming a fan-shaped structure, whereas the right one regressed. Gonadal asymmetry in female embryos was caused by differential expression of estrogen receptor (ER) gene in developing ovaries during ovarian development [59–61]. Additionally,* Pitx2* was reported to play a role on asymmetric ovarian development since its expression was only detected in the left ovary [62–64]. Besides a thickening of the cortex, the lacunae and fat-laden cells were observed in the left ovary. Previous studies reported that the lacunae are related to elimination of dead oogonia [65–67], whereas the fat-laden cells are associated with a steroid production [65]. Identification of the lacunae and fat-laden cells with special stains needs further study to verify specific cell type in these structures.

In conclusion, gonadogenesis in quail embryos is classified into three phases such as genital ridge formation, gonadal differentiation, and gonadal function based on embryonic gonadal development. Quail-PGCs were successfully detected in the genital ridges and the dorsal mesentery using WFA lectin histochemistry. Developing testes and ovaries can be distinguished histologically since day 8 of gonadal differentiation. The histology of embryonic testes was well delineated by Mallory-AZAN stain during gonadal function. This study provides new information regarding developmental biology and germ cell biology in avian species.

## Figures and Tables

**Table 1:** Quantification of WFA positive cells in the left and right genital ridges of 3-day-old quail embryos.

Number of stage 19 (P&I)* embryo (*n*)	^ +^WFA positive cells in the left gonadal ridges (mean ± SD)	WFA positive cells in the right gonadal ridges (mean ± SD)
1	141 (20 ± 4)	103 (15 ± 4)
2	211 (21 ± 6)	142 (14 ± 3)
3	294 (29 ± 7)	221 (22 ± 5)
4	230 (23 ± 3)	316 (32 ± 9)
5	336 (34 ± 5)	323 (32 ± 6)
6	284 (28 ± 4)	277 (28 ± 3)
Total	**1,496 (26 ± 7)**	**1,382 (24 ± 9)**

*Stage classified according to Padgett and Ivey, 1960 (P&I) [17].

^+^WFA (*Wisteria floribunda*) lectin binding with its sugar-binding specificity, *α*/*β*-GalNAc^¶^.

^¶^N-Acetylgalactosamine group.

The average PGC numbers (WFA positive cells) of quail embryos are indicated in parentheses.
